# Functional Properties of Human NMDA Receptors Associated with Epilepsy-Related Mutations of GluN2A Subunit

**DOI:** 10.3389/fncel.2017.00155

**Published:** 2017-05-29

**Authors:** Dmitry A. Sibarov, Nadine Bruneau, Sergei M. Antonov, Pierre Szepetowski, Nail Burnashev, Rashid Giniatullin

**Affiliations:** ^1^Laboratory of Comparative Neurophysiology, Sechenov Institute of Evolutionary Physiology and Biochemistry of the Russian Academy of SciencesSaint-Petersburg, Russia; ^2^INMED, Aix-Marseille University, INSERM U901Marseille, France; ^3^A. I. Virtanen Institute, University of Eastern FinlandKuopio, Finland; ^4^Laboratory of Neurobiology, Kazan Federal UniversityKazan, Russia

**Keywords:** GRIN2A, GluN2A, epilepsy, NMDA receptors, genetic variants

## Abstract

Genetic variants of the glutamate activated N-methyl-D-aspartate (NMDA) receptor (NMDAR) subunit GluN2A are associated with the hyperexcitable states manifested by epileptic seizures and interictal discharges in patients with disorders of the epilepsy-aphasia spectrum (EAS). The variants found in sporadic cases and families are of different types and include microdeletions encompassing the corresponding *GRIN2A* gene as well as nonsense, splice-site and missense *GRIN2A* defects. They are located at different functional domains of GluN2A and no clear genotype-phenotype correlation has emerged yet. Moreover, GluN2A variants may be associated with phenotypic pleiotropy. Deciphering the consequences of pathogenic *GRIN2A* variants would surely help in better understanding of the underlying mechanisms. This emphasizes the need for functional studies to unravel the basic functional properties of each specific NMDAR variant. In the present study, we have used patch-clamp recordings to evaluate kinetic changes of mutant NMDARs reconstituted after co-transfection of cultured cells with the appropriate expression vectors. Three previously identified missense variants found in patients or families with disorders of the EAS and situated in the N-terminal domain (p.Ile184Ser) or in the ligand-binding domain (p.Arg518His and p.Ala716Thr) of GluN2A were studied in both the homozygous and heterozygous conditions. Relative surface expression and current amplitude were significantly reduced for NMDARs composed of mutant p.Ile184Ser and p.Arg518His, but not p.Ala716His, as compared with wild-type (WT) NMDARs. Amplitude of whole-cell currents was still drastically decreased when WT and mutant p.Arg518His-GluN2A subunits were co-expressed, suggesting a dominant-negative mechanism. Activation times were significantly decreased in both homozygous and heterozygous conditions for the two p.Ile184Ser and p.Arg518His variants, but not for p.Ala716His. Deactivation also significantly increased for p.Ile184Ser variant in the homozygous but not the heterozygous state while it was increased for p.Arg518His in both states. Our data indicate that p.Ile184Ser and p.Arg518His GluN2A variants both impacted on NMDAR function, albeit differently, whereas p.Ala716His did not significantly influence NMDAR kinetics, hence partly questioning its direct and strong pathogenic role. This study brings new insights into the functional impact that *GRIN2A* variants might have on NMDAR kinetics, and provides a mechanistic explanation for the neurological manifestations seen in the corresponding human spectrum of disorders.

## Introduction

N-methyl-D-aspartate receptors (NMDARs) are cationic channels that are gated by the major excitatory neurotransmitter glutamate. They play a fundamental role in fast synaptic transmission, in temporal integration of neuronal network activity, and in synaptic plasticity. NMDAR-mediated signaling is involved in development, plasticity, learning, memory and high cognitive functions. NMDARs are composed of two obligatory GluN1 subunits and two modulatory subunits GluN2(A-D) or GluN3(A,B) to form either di- or tri-heterotetrameric channels (Paoletti et al., 2013). The identity of the GluN2 subunit is crucial for determining several gating properties, including maximal channel open probability, agonist sensitivity and deactivation kinetics; notably GluN1/GluN2A receptors have the fastest excitatory postsynaptic current decay, a key parameter in the control of synaptic integration.

There is long known association of NMDAR dysfunctions with various neurodevelopmental disorders (Kalia et al., 2008; Burnashev and Szepetowski, 2015). In 2013, a large number of heterozygous mutations were identified in the *GRIN2A* gene, which encodes the GluN2A subunit of NMDARs, in patients and families with disorders of the epilepsy-aphasia spectrum (EAS; Carvill et al., 2013; Lemke et al., 2013; Lesca et al., 2013). EAS disorders are childhood focal epilepsies and epileptic encephalopathies with speech and language dysfunction. They are characterized by onset of behavioral, language and cognitive impairment “acquired” during childhood after a period of apparently normal development, associated with (and likely influenced by) the presence of abundant interictal epileptiform discharges during sleep. Disease evolution and response to treatment are almost entirely unpredictable. So far usual therapeutic strategies rely on the administration of various antiepileptic drugs coupled with high-dose corticosteroids but overall success of the treatment remains unpredictable and unsatisfactory. One key challenge is whether more efficacious therapeutic interventions can be designed as early as possible in disease evolution to prevent against the most severe consequences.

The identification of *GRIN2A* defects as a first important cause for EAS disorders offers the potential for understanding pathogenic mechanisms and for testing treatment options. *In vitro* experiments could help in classifying the different GluN2A mutations and in understanding better both their specific and their shared effects. Coupled with the *in vitro* testing of various drugs such as NMDAR antagonists, this could even and ultimately lead to better selection of the appropriate therapeutic alternatives in the corresponding patients, as was proposed in pilot (Pierson et al., 2014) and subsequent studies (Swanger et al., 2016). Most GluN2A mutants studied to date have different or even opposing consequences on NMDAR properties, such as altered trafficking, loss of magnesium block, decreased or increased zinc inhibition, reduced proton sensitivity, decreased or increased glutamate potency, and/or slower kinetics (Carvill et al., 2013; Lemke et al., 2013; Lesca et al., 2013; Yuan et al., 2014; Serraz et al., 2016; Swanger et al., 2016; Ogden et al., 2017; for a review, see also Burnashev and Szepetowski, 2015). Whereas those studies have brought novel and important insights into the functional consequences of GluN2A mutations, effort is still needed to further decipher the different and complicated impacts of various *GRIN2A* mutations on the functioning of NMDARs.

To this aim, we studied the consequences of three different and previously discovered (Lesca et al., 2013) pathogenic mutations on the functioning of reconstituted NMDARs *in vitro*, in homozygous conditions as was previously done for most *GRIN2A* mutations studied so far, and in heterozygous conditions where—as in the human patients—both mutant and wild-type (WT) subunits are co-expressed within the same cells.

## Materials and Methods

### Constructs and Site-Directed Mutagenesis

The *GRIN1* cDNA construct (Genecopoeia EX-XO451-M61, thereafter designated as pGRIN1) was used for expression of WT human GluN1 receptor subunit (NP_000823) coupled with internal ribosome entry site (IRES)-driven independent expression of eGFP green fluorescent protein. Two *GRIN2A* cDNA constructs (Genecopoeia EX-T0084-M16 and EX-T0084-M56, thereafter designated as pGRIN2A-YFP and pGRIN2A-mCherry, respectively) were used for expression of WT human GluN2A receptor subunit (NP_000824) tagged with yellow fluorescent protein (YFP) or mCherry at the C-terminus, respectively. Mutant GluN2A constructs with mCherry at the C-terminus were generated from their WT counterpart by using QuikChange Lightning Site-Directed Mutagenesis Kit according to the manufacturer’s protocol (Agilent Technologies) and the following forward and reverse primers: GluN2A-Ile184Ser: 5′-CTGGCTACAGGGAATTCAGCAGCTTCGTCAAGACC and 5′-GGTCTTGACGAAGCTGCTGAATTCCCTGTAGCCAG (mutant Ile184Ser p.GRIN2A-mCherry); GluN2A-Arg518His: 5′-GCTCACCATCAATGAGGAACATTCTGAAGTGGTGG and 5′-CCACCACTTCAGAATGTTCCTCATTGATGGTGAGC (mutant Arg518His p.GRIN2A-mCherry); GluN2A-Ala716Thr: 5′-GAAAGGAGTAGAGGACACCTTGGTCAGCCTGAAAACGG and 5′-CCGTTTTCAGGCTGACCAAGGTGTCCTCTACTCCTTTC (mutant Ala716Thr p.GRIN2A-mCherry). The *GRIN1* and *GRIN2A* sequences from WT and mutant constructs were all verified by Sanger sequencing (GATC Biotech).

### Cell Cultures and Transfections

Human embryonic kidney (HEK) 293T cells were cultured as previously described (Qian et al., 2005). Cells were plated onto 7 mm glass coverslips treated with poly-L-lysine (0.2 mg/ml) in 35 mm culture dishes at 1 × 10^5^ cells per dish for patch clamp recordings, or in six-well plates at 2 × 10^5^ cells per well for immunocytochemistry experiments. Eighteen to twenty-four hours after plating, cells were transiently co-transfected with pGRIN1 and either of pGRIN2A-YFP or pGRIN2A-mCherry or both (see below), using FuGene HD reagent (Promega, WI, USA). Briefly, transfection was performed by adding to each dish 50 μl serum-free medium containing 1 μg total DNA and 2 μl FuGene. The combinations of plasmids used in this study are as follows: WT condition (GluN2A_WT): p.GRIN1+ WT p.GRIN2A-mCherry, ratio 1:4; p.Ile184Ser mutant homozygous condition (Ile184Ser_Hom): p.GRIN1+ mutant Ile184Ser p.GRIN2A-mCherry, ratio 1:4; p.Ala716Thr mutant heterozygous condition (Ile184Ser_Het): p.GRIN1+ mutant Ile184Ser p.GRIN2A-mCherry + WT p.GRIN2A-YFP, ratio 1:2:2; p.Ala716Thr mutant homozygous condition (Arg518His_Hom): p.GRIN1+ mutant Arg518His p.GRIN2A-mCherry, ratio 1:4; p.Arg518His mutant heterozygous condition (Arg518His_Het): p.GRIN1+ mutant Arg518His p.GRIN2A-mCherry + WT p.GRIN2A-YFP, ratio 1:2:2; p.Ala716Thr mutant homozygous condition (Ala716Thr_Hom): p.GRIN1+ mutant Ala716Thr p.GRIN2A-mCherry, ratio 1:4; p.Ala716Thr mutant heterozygous condition (Ala716Thr_Het): p.GRIN1+ mutant Ala716Thr p.GRIN2A-mCherry + WT p.GRIN2A-YFP, ratio 1:2:2. After incubation of cells for 6–8 h the transfection solution was replaced with fresh culture medium containing 200 μM DL-2-amino-5-amino-5-phosphono-valeric acid (DL-AP-5) and 2 mM Mg^2+^ to prevent against NMDAR-mediated excitotoxicity. The eGFP, mCherry and YFP fluorescent dyes were used to ascertain the efficacy of transfection assays and select the cells of interest for recordings or for membrane expression analysis.

### Patch Clamp Recordings

**Figure 1:**
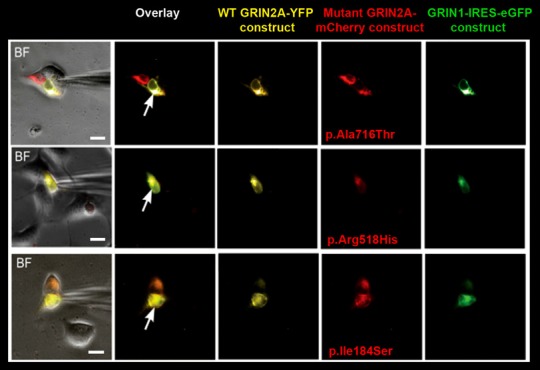
Selection of cells for whole-cell recordings. Triple transfection of human embryonic kidney (HEK) cells was performed with wild-type (WT) GRIN1-IRES-eGFP construct + WT GRIN2A-YFP construct + either of mutant GRIN2A-mCherry constructs (ratio 1:2:2). Cells with prominent fluorescence detection of all transfected subunits were selected for recordings of whole-cell currents (arrows). BF, bright field. Bar: 10 μm.

Whole-cell patch clamp recordings of membrane currents were performed 48–72 h after transfection of HEK293T cells expressing recombinant NMDARs with the appropriate subunit compositions (see above). Transfected cells were placed in extracellular solution of the following composition (in mM): NaCl 152, KCl 5, Glucose 10, HEPES 10, CalCl_2_ 2 (pH was adjusted to 7.3–7.4 with NaOH). Only cells with prominent fluorescence detection of all transfected subunits (two for homozygous conditions, three for heterozygous conditions) were selected (Figure 1). Excitation of fluorescence was made with Polychrom V monochromator with 488/10 nm for GFP, 514/10 for YFP and 585/10 for mCherry. Emission filters on fluorescent filter cubes were 510/20 for GFP, 560/50 for YFP and 625/30 for mCherry. Images were captured with PCO SensiCam digital camera. In case of successful recording a photo of cell fluorescence was obtained for eGFP, YFP and mCherry + brightfield (BF) to see electrode position. Recordings were made using a HEKA EPC10 USB patch-clamp amplifier controlled by HEKA PatchMaster software (HEKA Elektronik, Germany). The signal was 8–order low-pass Butterworth filtered at 20 Hz to remove high frequency noise. Acquisition rate was 100 s^−1^. Micropipette positioning was made with Sensapex micromanipulator (Sensapex, Finland) under visual control using Olympus IX50 microscope (Olympus, Japan). Patch pipettes (2–4 MΩ) were pulled from 1.5-mm (outer diameter) borosilicate standard wall capillaries with inner filament (Sutter Instrument, CA, USA). Pipette solution contained (in mM): CsCl_2_ 130, MgCl_2_ 5, HEPES 10, EGTA 5, CaCl_2_ 0.5, MgATP 2, NaGTP 0.5, KCl 5. pH was adjusted with CsOH to 7.2. Osmolarity was 300 mOsm. Experiments were performed at room temperature (23–25°C). After correction for the liquid junction potential between the Na^+^-containing external bathing solution and the Cs^+^-containing pipette solution of −15 mV, the default membrane voltage (*V*_m_) was set to −55 mV. Unless otherwise stated, to activate NMDARs we applied 1 mM glutamate with 10 μM glycine (Gly). Fast perfusion system (RSC-160 by BioLogic) was used for agonists application. The solutions exchange time estimated with open pipette was around 10 ms. Glutamate (Glu) was applied either for 0.1 s or for 10 s. The time constants of activation were estimated as single exponential approximation of a rise phase of response at the segment from 10% to 90% of amplitude. Deactivation time constants were estimated as weighted Tau of the double exponential fit of the current decay after 0.1 s Glu application. Desensitization time constants were estimated as weighted Tau of the double exponential fit of the current decay during 10 s Glu application. Decaying phase of the NMDAR-mediated currents was fitted with the sum of two exponentials:

$$I\left(t\right)=I_{\text{fast}}exp\left(-t/\tau_{fast}\right)+I_{\text{slow}}exp\left(-t/\tau_{\text{slow}}\right)$$

where *I*_fast_ and *I*_slow_ are the amplitudes of the fast and slow components, respectively, and *τ*_fast_ and *τ*_slow_ are the respective decay time constants. The weighted time constant of decays (*τ*_w_) was calculated using the following equation:

$$\tau_{\text{w}}=(I_{\text{fast}}/\text{​}\left(I_{\text{fast}}+I_{\text{slow}}\right))*\tau_{\text{fast}}+(I_{\text{slow}}/\text{​}\left(I_{\text{slow}}+I_{\text{fast}}\right))*\tau_{\text{slow}}$$

Current measurements were plotted and measured using either ClampFit 10.2 (Molecular Devices) or IgorPro softwares.

### Immunocytochemistry Experiments

Relative surface expression of GluN2A-mutant NMDARs was assessed in transfected HEK cells as compared with relative surface expression of WT NMDARs. Twenty-four hours after transfection, cells expressing the appropriate combination of NMDAR subunits (see above) were plated on coverslips coated with poly-D-Lysine in pre-warmed DMEM medium supplemented with 100 μM ketamine. Cells were washed twice with PBS and fixed with 4% paraformaldehyde (Antigenfix, Diapath) in PBS (pH 7.4) for 30 min at 4°C. To detect only the proportion of GluN2A-containing NMDARs that are located at the plasma membrane, immunostaining was done in non-permeabilized conditions. Briefly, cells were washed thrice, incubated 1 h at 4°C in PBS containing 3% bovine serum albumin (BSA), and then incubated overnight at 4°C with antibodies directed to the extracellular N-terminus of GluN2A (1:1000; Alomone) in PBS containing 3% BSA. Alexa fluor 647-conjugated anti-rabbit IgGs secondary antibodies (1/500; Thermo Fisher Scientific) were used. Nuclei were counterstained with Hoescht 33258 (1:2000, Sigma). Immunocytochemistry images were captured with a confocal microscope (LSM-800 Zeiss). The total cell and surface expressions of GluN2A-containing NMDARs were estimated by quantifying within the same cells Alexa fluor 647 and mCherry respectively, and using region of interest (ROI) manager of ImageJ software. The ratio of surface/total expression in each cell (expressed in arbitrary units, a.u.) were compared between each mutant combination and the WT combination of NMDAR subunits. Only the homozygous conditions were studied as the anti-GluN2A antibody would not discriminate between the respective surface expressions of WT and mutant GluN2A co-produced in the same cells.

### Data Analysis and Statistics

Quantitative data were expressed as means ± SEM unless otherwise stated. Number of experiments is indicated by *n* throughout. Sets of experiments were compared using Kruskal-Wallis test with Dunn’s multiple comparison post-test. Immunohistochemical data were considered significant based on a confidence level of 0.05. Electrophysiological data were considered significant based on a confidence level of 0.01 that takes into account Bonferroni’s correction for multiple testing (five parameters analyzed).

## Results

Three different GluN2A mutations situated in various parts of the NMDAR (Lesca et al., 2013) were studied. p.Ile184Ser is located in the leucine/isoleucine/valine-binding protein (LIVBP)-like N-terminal domain of GluN2A and was found in a patient with continuous spike and wave during slow sleep syndrome (CSWSS) and in his apparently unaffected mother. p.Arg518His occurs in the ligand-binding domain of GluN2A and caused disorders of the EAS with different degrees of severity in a single family: the daughter had CSWSS with verbal dyspraxia, her brother had atypical Rolandic epilepsy with verbal dyspraxia, and their father had verbal dyspraxia “only”. The same p.Arg518His mutation was also found in a patient with Landau-Kleffner syndrome (Conroy et al., 2014), which is another epileptic encephalopathy of the EAS. Last, p.Ala716Thr lies in another part of the ligand-binding domain and was inherited in seven members of a three-generation family who all presented with Rolandic epilepsy and verbal dyspraxia, and in one additional family member with verbal dyspraxia but no seizure.

### Surface Expression of Mutant NMDARs

**Figure 2:**
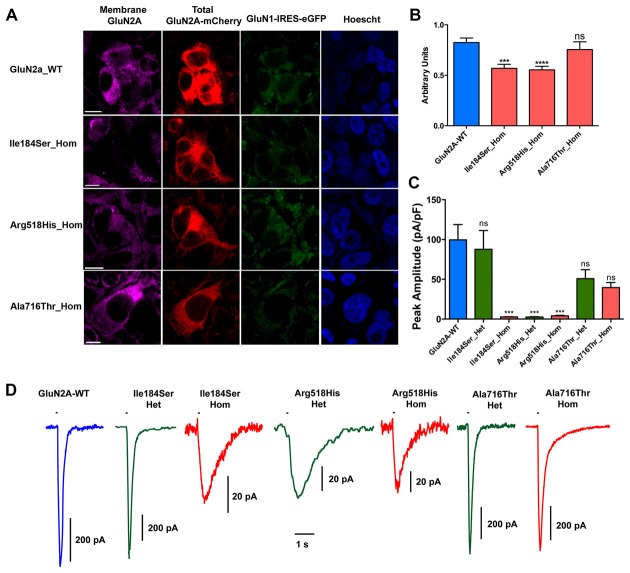
Relative surface expression and amplitude of whole-cell currents of NMDA receptors (NMDARs) with mutant GluN2A subunits. **(A)** HEK293T cells were cotransfected with the appropriate combinations of *GRIN1* and *GRIN2A* constructs for the expression of WT and of mutant recombinant NMDARs. *GRIN1* construct allowed for GluN1 subunit expression coupled with IRES-driven independent expression of eGFP fluorescent protein (green). *GRIN2A* constructs allowed for expression of GluN2A WT or mutant subunits fused to mcherry tag (red), which was used to estimate the total cell expression of GluN2A recombinant proteins. Surface expression of GluN2A-containing NMDARs was detected with antibodies directed to the N-terminus of GluN2A and with secondary antibodies coupled to Alexa-647 (magenta). Nuclei were counterstained with Hoescht (blue). Bar: 10 μm. Images were captured with confocal microscope. **(B)** Fluorescence intensities of Alexa-647 and of mCherry corresponding to the surface expression and to the total cell expression of GluN2A, respectively, were quantified within the same cells, using region of interest (ROI) manager of ImageJ software. Average relative surface expressions (RSE, ratio of surface to total expression) of WT (*n* = 63 cells) and of three mutant NMDARs (p.Ile184Ser, *n* = 46; p.Arg518His, *n* = 58; p.Ala718Thr, *n* = 27) are represented. RSE were significantly decreased for p.Ile184Ser and p.Arg518His, but not for p.Ala718Thr, as compared with WT (Table 1). Kruskall Wallis test followed by Dunn’s multiple comparison test. ****p < 0.0001; ***p < 0.001; ns: not significant. **(C)** Peak amplitudes of glutamate evoked whole-cell currents were normalized to the corresponding cell sizes and analyzed in NMDARs with WT and/or mutant GluN2A subunits (Table 1). p.Ile184Ser led to dramatic decrease of peak amplitude in homozygous but not in heterozygous condition. p.Arg518His led to dramatic decrease of peak amplitude in both the homozygous and heterozygous conditions. p.Ala716Thr had no significant effect on peak amplitude in either of homozygous or heterozygous conditions. Kruskal-Wallis test with Dunn’s multiple comparison test. ***p < 0.001; ns: not significant. **(D)** Representative example traces of whole-cell currents for WT and/or mutant GluN2A subunits evoked by 100 ms pulses of 1 mM glutamate (bar above each trace) illustrating reliability of the recordings for the NMDAR mutants with small amplitude. Note that amplitude scale bars shown near each trace are different. Time scale bar applies for all traces.

First, we tested whether membrane trafficking of GluN2A subunits would be altered by the missense mutations studied here. To this aim, relative surface expression (RSE: ratio of surface to total expression) of WT and either of three mutant NMDARs reconstituted in HEK cells were measured by fluorescence-based immunocytochemistry and computer-assisted quantitative image analysis. RSE was significantly reduced for NMDARs composed of mutant p.Ile184Ser (Ile184Ser_Hom) (RSE = 0.57 ± 0.04 a.u., *p* = 0.0001; *n* = 46 cells) and of mutant p.Arg518His (Arg518His_Hom) (RSE = 0.55 ± 0.04 a.u., *p* < 0.0001; *n* = 58), as compared with WT NMDARs (RSE = 0.83 ± 0.05 a.u.; *n* = 63). In contrast, no significant change in surface expression was seen with mutant p.Ala716Thr (Ala716Thr_Hom) (RSE = 0.76 ± 0.08 a.u.; *n* = 27) (Figures 2A,B; Table 1).

**Table 1:** Summary of data on wild-type and mutant N-methyl-D-aspartate receptors (NMDARs).

NMDARs	WT	Ile184Ser_Het	Ile184Ser_Hom	Arg518His_Het	Arg518His_Hom	Ala716Thr_Het	Ala716Thr_Hom
Surface expression (a.u.)	0.82 ± 0.04 (*n* = 63)	ND*	0.57 ± 0.04 (*n* = 46) *p* = 0.0001	ND*	0.55 ± 0.04 (*n* = 58) *p* < 0.0001	ND*	0.76 ± 0.08 (*n* = 27) *p* = 0.7284
Amplitude (pA/pF)	99.6 ± 19.2 (*n* = 20)	87.8 ± 23.5 (*n* = 10) *p* > 0.9999	2.6 ± 0.3 (*n* = 19) *p* < 0.0001	2.5 ± 0.4 (*n* = 16) *p* < 0.0001	4.1 ± 0.6 (*n* = 16) *p* < 0.0001	50.8 ± 11.2 (*n* = 9) *p* > 0.9999	39.5 ± 6.4 (*n* = 11) *p* > 0.9999
*τ*_A_ (ms): Activation time constant	42.8 ± 2.5 (*n* = 10)	168.3 ± 20.0 (*n* = 31) *p* < 0.0001	105.8 ± 10.8 (*n* = 37) *p* = 0.0062	156.4 ± 10.6 (*n* = 28) *p* < 0.0001	153.1 ± 17.3 (*n* = 36) *p* < 0.0001	79.1 ± 12.2 (*n* = 9) *p* = 0.4752	81.4 ± 10.7 (*n* = 18) *p* = 0.2478
*τ*_D_ (ms): Deactivation time constant	136.2 ± 11.5 (*n* = 10)	158.1 ± 8.9 (*n* = 11) *p* > 0.9999	652.9 ± 49.7 (*n* = 17) *p* < 0.0001	831.4 ± 29.9 (*n* = 8) *p* < 0.0001	492.5 ± 39.5 (*n* = 14) *p* = 0.0008	130.8 ± 20.5 (*n* = 8) *p* > 0.9999	371.9 ± 27.5 (*n* = 11) *p* = 0.0481
*τ*_DES_ (ms): Desensitization time constant	2427 ± 488 (*n* = 9)	1740 ± 233 (*n* = 9) *p* = 0.7503	NM	NM	NM	1983 ± 274 (*n* = 7) *p* > 0.9999	2482 ± 299 (*n* = 10) *p* > 0.9999
*I*_SS_/*I*_Peak_: Steady-state to peak relation	0.44 ± 0.06 (*n* = 9)	0.45 ± 0.04 (*n* = 9) *p* > 0.9999	0.81 ± 0.02 (*n* = 5) *p* = 0.0181	0.71 ± 0.06 (*n* = 5) *p* = 0.1161	0.82 ± 0.02 (*n* = 8) *p* = 0.0025	0.37 ± 0.04 (*n* = 8) *p* > 0.9999	0.49 ± 0.03 (*n* = 11) *p* > 0.9999

a.u., arbitrary units; ms, millisecond; ND, not done; NM, not measurable; ns, not significant; pA, picoAmpere; pF, picoFarrad; s, second; WT, wild-type. Number of cells analyzed for each parameter are indicated in brackets. p values are before Bonferroni correction. * Surface expression was not measured in the heterozygous conditions as the anti-GluN2A antibody would not discriminate between the respective contribution of wild-type and mutant GluN2A co-expressed in the same cells ns, not significant: p > 0.05 for surface expression data; p > 0.01 for all five electrophysiological parameters.

### Amplitude of Whole-Cell Currents in Mutant NMDARs

Next, we analyzed peak amplitudes of the whole-cell currents in WT and mutant NMDARs (Figures 2C,D; Table 1). Consistent with the data of surface expression presented above, peak current amplitudes in the homozygous conditions were significantly lower for Ile184Ser_Hom (2.6 ± 0.3 pA/pF, *p* < 0.0001; *n* = 19 cells) and Arg518His_Hom (4.1 ± 0.6 pA/pF, *p* < 0.0001; *n* = 16), but not for Ala716Thr_Hom (39.5 ± 6.4 pA/pF; *n* = 11), as compared with the WT (99.6 ± 19.2 pA/pF; *n* = 20). In the heterozygous conditions, peak amplitudes of whole-cell currents were not significantly different from WT, not only for Ala716Thr_Het condition (50.8 ± 11.2 pA/pF; *n* = 9) as expected from the data in homozygous condition, but also for Ile184Ser_Het (87.8 ± 23.5 pA/pF; *n* = 10). In sharp contrast, peak amplitude of whole-cell currents still remained drastically decreased at a level similar to the corresponding mutant homozygous condition (2.5 ± 0.4 pA/pF, *p* < 0.0001; *n* = 16) when WT and mutant p.Arg518His GluN2A subunits were co-expressed (Arg518His_Het) in the same cells. Thus, our data suggested that in the homozygous condition, two of the three mutant NMDARs (p.Ile184Ser and p.Arg518His GluN2A subunits) were poorly expressed at membrane level, whereas, in the heterozygous condition, sufficient membrane expression of NMDARs with p.Arg518His GluN2A subunit could still be associated with reduced responsiveness to agonist.

### Activation and Deactivation Kinetics of NMDARs

**Figure 3:**
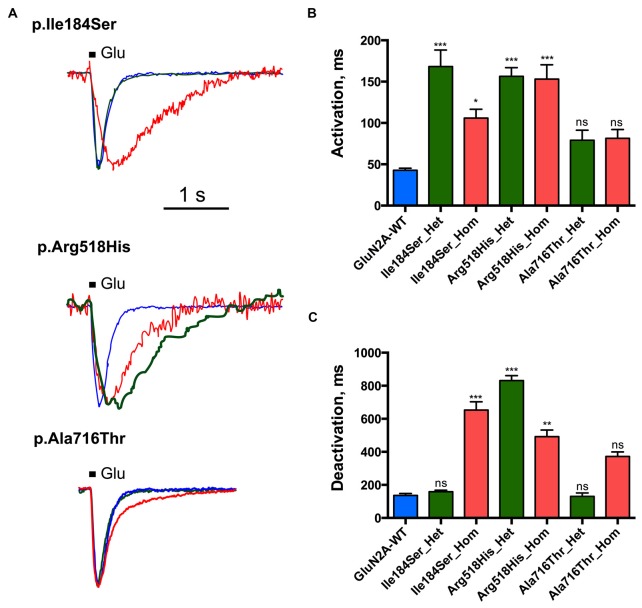
Activation and deactivation properties of NMDARs with mutant GluN2A subunits. Whole-cell patch clamp recordings of membrane currents were performed on HEK293T cells cotransfected with the appropriate combinations of *GRIN1* and *GRIN2A* constructs for the expression of WT and/or of mutant recombinant NMDARs. **(A)** An overlay of currents evoked by 100 ms Glu application in the WT (blue), homozygous (red) and heterozygous (green) conditions. Currents are normalized by peak amplitudes. **(B)** Averaged activation time constants. p.Ile184Ser and p.Arg518His led to significant increases of activation time constant in both homozygous and heterozygous conditions. p.Ala716Thr had no significant effect on activation time constant in either homozygous or heterozygous conditions. Kruskal-Wallis test with Dunn’s multiple comparison test. ***p < 0.0002; *p < 0.01; ns: not significant. **(C)** Averaged deactivation time constants. p.Ile184Ser led to dramatic increase of deactivation time constant in homozygous but not in heterozygous condition. p.Arg518His led to significant increase of deactivation time constant in both homozygous and heterozygous conditions. p.Ala716Thr had no significant effect on deactivation time constant in either of homozygous or heterozygous conditions. Kruskal-Wallis test with Dunn’s multiple comparison test. ***p < 0.0002; **p < 0.002; ns, not significant.

We then tested whether key kinetic parameters of NMDARs such as activation and deactivation were affected by these missense mutations. To this end, we first measured the rising and decay phases of the WT and homozygous mutant NMDARs (Figures 3A,B; Table 1). Homozygous mutant receptors with p.Ile184Ser and p.Arg518His GluN2A variants yielded activation time constants (*τ*_A_) that were significantly slower than that of WT receptors: *τ*_A_ was 42.8 ± 2.5 ms for WT (*n* = 10 cells); 105.8 ± 10.8 ms for Ile184Ser_Hom (*n* = 37, *p* = 0.006 vs. WT); and 153.1 ± 17.3 ms for Arg518His_Hom (*n* = 36, *p* < 0.0001 vs. WT). In contrast, *τ*_A_ did not change significantly for receptors with p.Ala716Thr mutant GluN2A subunits (Ala716Thr_Hom) (81.4 ± 10.7 ms; *n* = 18). Similar to the homozygous conditions, activation times were also significantly decreased in heterozygous conditions for the two p.Ile184Ser and p.Arg518His mutations but not for the p. Ala716Thr mutation (Figures 3A,B; Table 1).

Deactivation time constant (*τ*_D_) was 136.2 ± 11.5 ms (*n* = 10 cells) for WT NMDARs (Figures 3A,C; Table 1). This parameter significantly increased for p.Ile184Ser mutation in the homozygous state (652.9 ± 49.7 ms, *n* = 17, *p* < 0.0001 vs. WT) but not in the heterozygous state (158.1 ± 8.9 ms, *n* = 11). *τ*_D_ was prolonged for p.Arg518His in both the homozygous (492.5 ± 39.5 ms, *n* = 14, *p* = 0.0008 vs. WT) and heterozygous (831.4 ± 29.9 ms, *n* = 8, *p* < 0.0001 vs. WT) conditions. Finally, *τ*_D_ was also increased by the p.Ala716Thr mutation in the homozygous state (371.9 ± 27.5 ms, *n* = 11) but statistical trend towards significance (*p* = 0.048) did not resist correction for multiple testing. In the heterozygous Ala716Thr/WT state *τ*_D_ was not significantly modified either (130.8 ± 20.5 ms, *n* = 8).

### Desensitization of NMDARs

**Figure 4:**
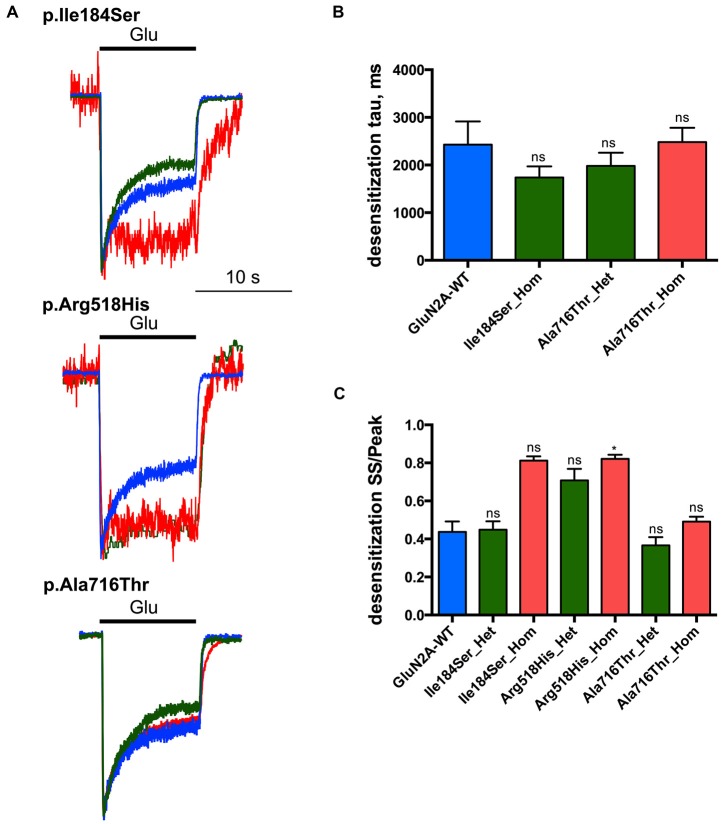
Desensitization properties of NMDARs with mutant GluN2A subunits. Whole-cell patch clamp recordings of membrane currents were performed on HEK293T cells cotransfected with the appropriate combinations of *GRIN1* and *GRIN2A* constructs for the expression of WT and/or of mutant recombinant NMDARs. **(A)** An overlay of currents evoked by 10 s Glu application in the WT (blue), homozygous (red) and heterozygous (green) conditions. Currents are normalized by peak amplitudes. **(B)** Averaged desensitization time constants (*τ*_DES_). No significant change in *τ*_DES_ was detected for any condition tested. Of note, *τ*_DES_ was not measurable for p.Ile184Ser in the homozygous condition, and for p.Arg518His in homozygous and heterozygous conditions because of nearly absent desensitization (Table 1). Kruskal-Wallis test with Dunn’s multiple comparison test. ns: not significant. **(C)** Averaged steady-state to peak relation (*I*_END10s_/*I*_P_ ratio). p.Ile184Ser and p.Ala716Thr had no significant effect on *I*_END10s_/*I*_P_ ratio in either of homozygous or heterozygous conditions. p.Arg518His led to significant increase *I*_END10s_/*I*_P_ ratio in homozygous but not in heterozygous condition. Kruskal-Wallis test with Dunn’s multiple comparison test. *p < 0.01; ns, not significant.

Next we tested whether NMDAR mutations would also affect desensitization, which in turn would affect neuronal excitability *in vivo*. This phenomenon was quantified using two parameters: (i) desensitization time constant (*τ*_DES_), which characterizes desensitization onset (Figures 4A,B; Table 1); and (ii) the ratio of the current amplitude measured at the end of a 10 s glutamate pulse to the peak amplitude (*I*_END10s_/*I*_P_), which characterizes the degree of desensitization within the 10 s period (Figures 4A,C; Table 1). For WT NMDARs, *τ*_DES_ was 2427 ± 488 ms and *I*_END10s_/*I*_P_ ratio was 0.44 ± 0.06 (*n* = 9 cells). None of the two parameters showed significant modification for the p.A716T mutation either in homozygous (*τ*_DES_: 2482 ± 299 ms, *n* = 10; *I*_END10s_/*I*_P_: 0.49 ± 0.03, *n* = 11) or in heterozygous (*τ*_DES_: 1983 ± 274 ms, *n* = 7; *I*_END10s_/*I*_P_: 0.37 ± 0.04, *n* = 8) conditions. NMDARs bearing the p.Arg518His mutation showed *I*_END10s_/*I*_P_ ratio close to 1 in either homozygous (0.82 ± 0.02, *n* = 8) or heterozygous (0.71 ± 0.06, *n* = 5) states; this was indeed statistically significant in the homozygous state (*p* = 0.0025). Similarly, p.Ile184Ser showed poor desensitization in the homozygous state, with *I*_END10s_/*I*_P_ at 0.81 ± 0.02 (*n* = 5); however statistical trend towards significance (*p* = 0.0181) did not resist Bonferroni correction. Because of nearly absent desensitization, *τ*_DES_ was not measurable for these conditions. In contrast, desensitization parameters were not modified in the Ile184/WT condition (*τ*_DES_: 1740 ± 233 ms, *n* = 9; *I*_END10s_/*I*_P_: 0.45 ± 0.04, *n* = 9).

## Discussion

The three variants that were studied here and that are located in the extracellular part of GluN2A subunit, either in its N-terminal domain (p.Ile184Ser) or in its ligand-binding domain (p.Arg518His and p.Ala716Thr), differently impacted on the kinetics properties of NMDARs *in vitro*. Complicated and even opposing consequences of several GluN2A mutations on the functional properties of NMDARs, have already been reported (Burnashev and Szepetowski, 2015; Serraz et al., 2016; Swanger et al., 2016). Two of the three variants studied here, p.Ile184Ser and p.Arg518His, showed dramatically reduced current responses to 1 mM glutamate and 10 μM glycine when analyzed in the homozygous state; in line with this, those two variants also led to reduced surface expression of the corresponding mutant NMDARs, by 31% and 33%, respectively. In addition to reduced membrane trafficking, those two variations are likely to cause functional changes of the mutant NMDARs, as the decreases in current responses were much more pronounced (p.Ile184Ser: 97.4% reduction; p.Arg518His: 95.9%). In a previous publication (Serraz et al., 2016), p.Ile184Ser apparently also led to decreased membrane expression but no change in current amplitude was observed. This discrepancy with our study might be due to differences in the methodologies used, as these authors used *Xenopus* oocytes as a heterologous expression system whereas human HEK cells were used in the present study. Moreover, reduced surface expression and much more dramatic decrease in current amplitude of NMDARs as detected here with p.Arg518His-mutant homozygous GluN2A, are consistent with recent observations showing approximately 50% reduction in membrane expression and nearly no current response (Swanger et al., 2016). The 54% decrease in current amplitude previously associated with p.Ala716Thr (Swanger et al., 2016) was also somehow replicated here in both the homozygous (60% reduction) and heterozygous (49%) conditions, but this reduction was not statistically significant. Moreover, the decrease in surface expression of NMDARs caused by p.Ala716Thr as reported previously (Swanger et al., 2016), was not found here. This might be due to the two different methods used in this and in our study, respectively, to quantify plasma membrane expression. It should also be mentioned that the experiments were performed *in vitro* and the respective contributions of the WT and mutant subunits to the composition of NMDARs might be different in the patients’ cells. Also, the possible impact of GluN2A variants on the functioning of tri-heteromeric NMDARs was not studied here.

It was reported that regardless of close location to zinc binding site of GluN2A, p.Ile184Ser does not significantly influence either EC_50_ of glutamate or glycine binding, or zinc sensitivity of NMDARs (Serraz et al., 2016). In addition to the aforementioned reduced plasma membrane expression and low current amplitude, homozygous p.Ile184Ser was also associated with changes in kinetics properties, leading to a 4.8-fold increase in deactivation time constant and a 2.5-fold increase in activation time constant, while leaving desensitization parameters unchanged. p.Ile184Ser was not reported to interact with NMDAR modulators like zinc, protons or spermine; p.Ile184Ser is not situated in the ligand binding domain and slows down receptor deactivation by an as-yet unknown mechanism. In the heterozygous condition p.Ile184Ser-related changes were no longer seen, with the noticeable exception of receptor activation, which looked even more pronounced in the heterozygous than in the homozygous state. This suggested that NMDAR activation could be impacted by the p.Ile184Ser GluN2A variant in the heterozygous state at the cellular level.

The Arg518 residue is fully conserved between GluA, GluN, GluK and GluD receptors (Traynelis et al., 2010). Glutamate binding to GluN2A involves interactions of the agonist alpha-carboxylate group with Arg518 (Furukawa et al., 2005). Here we show that p.Arg518His variation led to very low amplitude currents with slow activation and deactivation kinetics. Slow activation speed probably prevents the first peak in response to fast glutamate application. Deactivation was also slow, which means longer period of ligand bound state of receptor, which in turn probably results in very low amplitude of whole cell currents and increases the duration of receptor open state. Noteworthy, the prolonged deactivation of current response to 100 ms glutamate application was consistent with that of simulated currents based on single channel properties of p.Arg518His-GluN2A NMDARs as reported previously (Lesca et al., 2013). Surprisingly, most functional changes of NMDARs caused by p.Arg518His were detected in both homo- and heterozygous conditions, at similar (activation kinetics) or even higher (current amplitude, deactivation kinetics) rates, suggesting that p.Arg518His might act in a dominant-negative way on those parameters. This interpretation has to be taken with caution as it relies on *in vitro* experiments; moreover the actual stoichiometry of the mutant and WT GluN2A subunits within each transfected cell, and the actual subunit composition of each NMDAR present at the cell surface, are not known. Nevertheless a possible partial dominant-negative effect that would be exerted *in vivo* on NMDAR kinetics, and the impact of p.Arg518His on other NMDAR properties that were not studied here, would probably underlie comparable phenotypes and degrees of severity as those caused by heterozygous *GRIN2A* haploinsufficiency defects (e.g., microdeletions). Dominant-negative effects on some NMDAR properties had already been observed for the p.Leu812Met GluN2A variant that was studied in NMDARs composed of one (heterozygous state) or two (homozygous state) GluN2A variants at the cell surface (Yuan et al., 2014). For the other GluN2A variants studied in heterozygous mutant NMDARs as compared with their homozygous counterparts, intermediate effects consistent with haploinsufficiency were seen (Endele et al., 2010; Lemke et al., 2013; Gao et al., 2017; Ogden et al., 2017). However most GluN2A variants were studied in the homozygous state only. Hence our findings on p.Arg518His emphasize the need for more systematic analysis of GluN2A variants in both homozygous and heterozygous situations in order to discriminate better between different possible mechanisms. It was also suggested that similar disease phenotypes could result from both enhanced and reduced NMDAR function (Swanger et al., 2016). Whatever the underlying mechanisms, they do not solely account for the diversity of the phenotypes caused by p.Arg518His, from the not-so-severe verbal dyspraxia to the epileptic encephalopathy known as CSWSS in a single family (Lesca et al., 2013).

The Ala716 residue is located in the S2 segment of the ligand binding domain and does not interact directly with glutamate. In contrast with the two other variants studied here, p.Ala716Thr-GluN2A had weaker effect on NMDAR functioning, at least according to the parameters that were analyzed: hence surface expression and desensitization parameters were similar to control values, and the changes in current amplitude did not reach statistical significance. Similarly the trend towards increased deactivation time constant of homozygous mutant NMDARs did not resist correction for multiple testing. It was recently shown that p.Ala716Thr had a moderate effect on surface expression, current amplitude and deactivation, and a nearly 10-fold decrease in glutamate potency (Swanger et al., 2016). Whereas those discrepancies might be due to various factors, such as the different constructs used to drive NMDAR subunit expressions, or even the statistical thresholds and corrections used for multiple testing, it is interesting to note that p.Ala716Thr variant was found in patients of a family with Rolandic epilepsy and verbal dyspraxia who all co-inherited a rare and dominant-negative genetic variant in the Sushi-Repeat containing protein SRPX2 (Roll et al., 2006; Salmi et al., 2013), hence raising the possibility that the phenotypes would be caused by those two GluN2A and SRPX2 variants acting together.

Overall our data bring additional insights into the functional impact that *GRIN2A* variants might have, notably on NMDAR kinetics. Of the three variants studied here, one had weak effects, if any, and the two others had different dominant-negative and haploinsufficiency consequences. Effort is now needed to understand how the numerous *GRIN2A* variants and defects and their complicated functional consequences lead to the neurological manifestations seen in the corresponding human spectrum of disorders.

## Author Contributions

DAS and NBr performed the experiments and data analyses with equal contributions. SMA participated in electrophysiological experiments. NBu participated in and supervised electrophysiological data analysis. PS, NBu and RG decided on the overall strategy and directed the follow-up of experiments with equal contributions. PS, NBu and RG wrote the manuscript with help of DS and NBr.

## Conflict of Interest Statement

The authors declare that the research was conducted in the absence of any commercial or financial relationships that could be construed as a potential conflict of interest.
